# Gene and protein profiling of Dickkopf-3 and complement factor H in periodontitis and coronary artery disease

**DOI:** 10.3389/fdmed.2025.1679757

**Published:** 2025-11-20

**Authors:** Jaideep Mahendra, Bawatharani Maharavi, Prabhu Manickam Natarajan, Moongilpatti Arumugam

**Affiliations:** 1 Department of Periodontics, Meenakshi Ammal Dental College and Hospital, Chennai, India; 2 Department of Clinical Sciences, Center of Medical and Bio-Allied Health Sciences and Research, College of Dentistry, Ajman University, Ajman, United Arab Emirates; 3 Department of Cardiology, Kilpauk Medical College and Hospital, Chennai, India

**Keywords:** coronary artery disease, periodontitis, molecular biology, periodontal medicine, Dickkopf-3, complement factor H

## Abstract

**Background:**

This study aimed to evaluate and compare demographic, periodontal, and cardiac parameters; Dickkopf-3 (DKK-3; rs11544817) and complement factor H (CFH; rs10737680) protein levels; and gene expression in patients with periodontitis with and without coronary artery disease (CAD) at baseline and following non-surgical periodontal therapy (NSPT).

**Methods:**

A total of 140 participants were divided into the following four groups (*n* = 35 each): Group H, healthy individuals; Group P, periodontitis patients without systemic conditions; Group CAD, systemically compromised (CAD) but periodontally healthy individuals; and Group P + CAD, patients with both periodontitis and CAD. The baseline assessments included demographic details, periodontal and cardiac parameters, and protein/gene analysis from subgingival plaque and gingival tissue. NSPT was performed and assessments were conducted after 90 days.

**Results:**

Group P + CAD had higher age, weight, and BMI, and lower socioeconomic status. Periodontal and cardiac parameters improved after NSPT. DKK-3 levels decreased and CFH levels increased post-treatment. Both proteins strongly correlated with gene expression, independent of confounders. The regression analysis confirmed their significant association with periodontitis and CAD risk.

**Conclusion:**

The study found elevated DKK-3 (rs11544817) and reduced CFH (rs10737680) protein levels and gene expression in patients with periodontitis and CAD, suggesting that these genes regulate their respective proteins.

## 1 Introduction

Periodontitis is a common chronic bacterial infection that causes destruction to the supporting structures of the teeth, leading to tissue destruction and potential alveolar bone loss. Its progression is multifactorial and influenced by local factors such as plaque, genetics, systemic health, pregnancy, and lifestyle, with the host response determining disease severity. The immune system is vital in defending against periodontal infections and promoting tissue repair. While topical or oral drugs can help treat bacterial infections, non-surgical mechanical debridement remains the gold standard for eradicating periodontal infections and ensuring better periodontal health. Periodontal pathogens, cytokines, and pro-inflammatory mediators can enter systemic circulation, causing transient bacteremia and toxemia, which may contribute to conditions such as atherosclerotic cardiovascular disease, diabetes, adverse pregnancy outcomes, respiratory diseases, chronic kidney disease, and rheumatoid arthritis (1, 2).

Early detection and effective management of periodontitis are essential not only to preserve oral health but also to prevent associated systemic conditions. Although clinical and radiographic assessments remain foundational diagnostic tools, biomarkers have emerged as vital adjuncts for early diagnosis, monitoring disease activity, predicting progression, and evaluating therapeutic outcomes. These biomarkers can be derived from various biological fluids and tissues, including urine, blood, saliva, gingival crevicular fluid (GCF), dental plaque, and gingival tissues, and they serve diverse functions as diagnostic, prognostic, and risk assessment indicators (3). Several pro-inflammatory and bone-regulatory molecules, such as interleukin (IL)-1β, IL-6, IL-8, IL-17, C-reactive protein (CRP), matrix metalloproteinases (MMPs), and receptor activator of nuclear factor kappa-Β ligand (RANKL), have been extensively investigated as biomarkers in periodontitis (4). These mediators not only reflect local periodontal inflammation and tissue destruction but also contribute to the systemic inflammatory burden, thereby establishing a potential pathogenic link between periodontitis and cardiovascular diseases.

Dickkopf (DKK), a glycoprotein, is emerging as a diagnostic biomarker in various inflammatory diseases. DKK consists of four members, namely, DKK-1, DKK-2, DKK-3, and DKK-4. These glycoproteins regulate the wingless-related integration site (Wnt) and transforming growth factor beta (TGF-β) signaling pathways (5). Among the DKK proteins, DKK-3 has recently emerged as an important player in the development of atherosclerotic plaque and participates in different stages of atherosclerosis, from endothelial dysfunction to lipid deposit and from initial inflammation to plaque formation. A large number of studies have shown that serum DKK-3 levels are closely associated with atherosclerotic diseases, such as early myocardial infarction and ischemic cerebrovascular disease (6, 7). Hence, DKK-3 has recently gained attention as a potential biomarker for cardiovascular disease, particularly in assessing atherosclerosis risk.

DKK proteins act as Wnt antagonists and inhibit osteoblast differentiation, ultimately leading to bone resorption. Elevated DKK levels have been associated with bone loss and impaired osteogenesis, whereas reduced levels promote bone formation. Although not yet investigated in the context of periodontitis, DKK-3 has gained recognition as a key biomarker in cardiovascular diseases, particularly atherosclerosis. The chronic inflammation and bone-destructive mechanisms seen in periodontitis may elevate systemic levels of inflammatory mediators, such as DKK-3, contributing to endothelial dysfunction, vascular inflammation, and plaque formation. This shared inflammatory axis suggests a mechanistic link between periodontitis and cardiovascular disease, highlighting the importance of DKK-3 as one of the significant markers for coronary artery disease (CAD) and periodontitis (8).

In contrast, complement factor H (CFH) is a glycoprotein that regulates the alternative complement pathway, preventing host tissue damage while targeting pathogens. Synthesized mainly in the liver, it controls the complement system to protect healthy cells and limit damage during inflammation and disease. CFH accelerates the alternative C3 convertase through competitive binding with C3b and its inadequate recognition of cells can cause conditions such as atypical hemolytic uremic syndrome (aHUS), age-related macular degeneration (ARMD), and membrano-proliferative glomerulonephritis type II. CFH polymorphisms are also linked to an increased risk of inflammatory diseases, including cardiovascular and periodontal diseases, particularly in the elderly (9). There is an increasing number of studies linking biomarkers of the subgingival microbiome in periodontal diseases with systemic conditions; nevertheless, research on the gene expression of specific biomarkers remains limited. The DKK-3 and CFH proteins have been associated with inflammatory diseases. However, the gene expression of DKK-3 (rs11544817) and CFH (rs10737680) and their protein levels in patients with periodontitis and CAD following non-surgical periodontal therapy (NSPT) have not been explored. This novel study aims to assess the gene expression and protein level changes of both the above glycoproteins in CAD and periodontitis and compare them with periodontal parameters, thus evaluating their role as potential biomarkers in early diagnosis and harnessing their therapeutic potential in linking periodontal disease and CAD.

## 2 Materials and methods

### 2.1 Study design

This study was conducted from June 2023 to November 2024 in Chennai, Tamil Nadu, India, and was carried out in accordance with the 1975 Helsinki Declaration, as revised in 2013. The study was approved by the Institutional Ethical Committees of both Meenakshi Ammal Dental College and Hospital and Kilpauk Medical College and Hospital, Chennai (MADC/IEC-I/3/2023 and 1035/2023, respectively), and was registered with ClinicalTrials.gov (ID: NCT05828368), ensuring compliance with all relevant ethical standards. Written informed consent was obtained from all participants.

The inclusion criteria were as follows: (1) willingness to participate; (2) aged 30–65 years; (3) male or female; (4) varying CAD types [i.e., stable angina, unstable angina, ST-elevation myocardial infarction (STEMI), or non-ST-elevation myocardial infarction (NSTEMI)] and severities; (5) ≥20 natural teeth; (6) a comprehensive full-mouth clinical examination of all teeth, including third molars, with the third molars evaluated for inclusion based on specific criteria, such as being fully erupted and accessible for a clinical examination that included probing depth and clinical attachment loss (CAL). Patients with (1) partially erupted teeth or who had unique conditions that may not have represented generalized periodontal health or disease (e.g., pericoronitis) were excluded, as were those with (2) systemic conditions such as diabetes, respiratory disorders, renal and liver diseases, rheumatoid arthritis, malignancies, and HIV; (3) those who used corticosteroids, antibiotics (within 6 months), or antiepileptic drugs; (4) those who were pregnant; (5) current or recent smokers (who quit smoking <6 months); and (6) those with a history of periodontal therapy in the past 6 months. The participants were carefully selected based on the above criteria to ensure a balanced and comparable study population, thereby maintaining the integrity and reliability of the study outcomes. Both the healthy (H) and periodontitis (P) groups were selected from the Department of Periodontics, Meenakshi Ammal Dental College and Hospital. The H group comprised healthy individuals characterized by the following: no CAL of ≥1 mm, except for cases with non-periodontitis-related CAL (e.g., traumatic recession); no bleeding on probing (BOP) at <10% of sites; pocket depth (PD) of ≤3 mm at all sites; no radiographic evidence of bone loss; had attended regular oral health checkups; and without any oral and systemic diseases. The P group comprised individuals with 30% or more of sites having a CAL of ≥3 mm (for Stage II) or ≥5 mm (for Stage III), radiographic evidence of alveolar crestal bone loss extending ≥15% from the cementoenamel junction, and no systemic diseases, as classified based on the 2017 World Workshop for the Classification of Periodontal and Peri-Implant Diseases and Conditions (10).

Moreover, the patients in the CAD group and periodontitis and coronary artery disease (P + CAD) group were recruited from the Department of Cardiology, Kilpauk Medical College and Hospital, Chennai. In these patients, CAD was diagnosed by a cardiologist based on the following criteria: clinical history, symptoms, ECG findings, angiography showing ≥50% stenosis, stress testing with positive results indicating myocardial ischemia or infarction, and cardiac imaging showing evidence of prior myocardial infarction or reduced ejection fraction on echocardiography or other imaging modalities. Myocardial infarction was identified in line with the Fourth Universal Definition (2018), requiring elevated troponin with ischemic symptoms, ECG changes, or imaging evidence (11).

**Figure 1:**
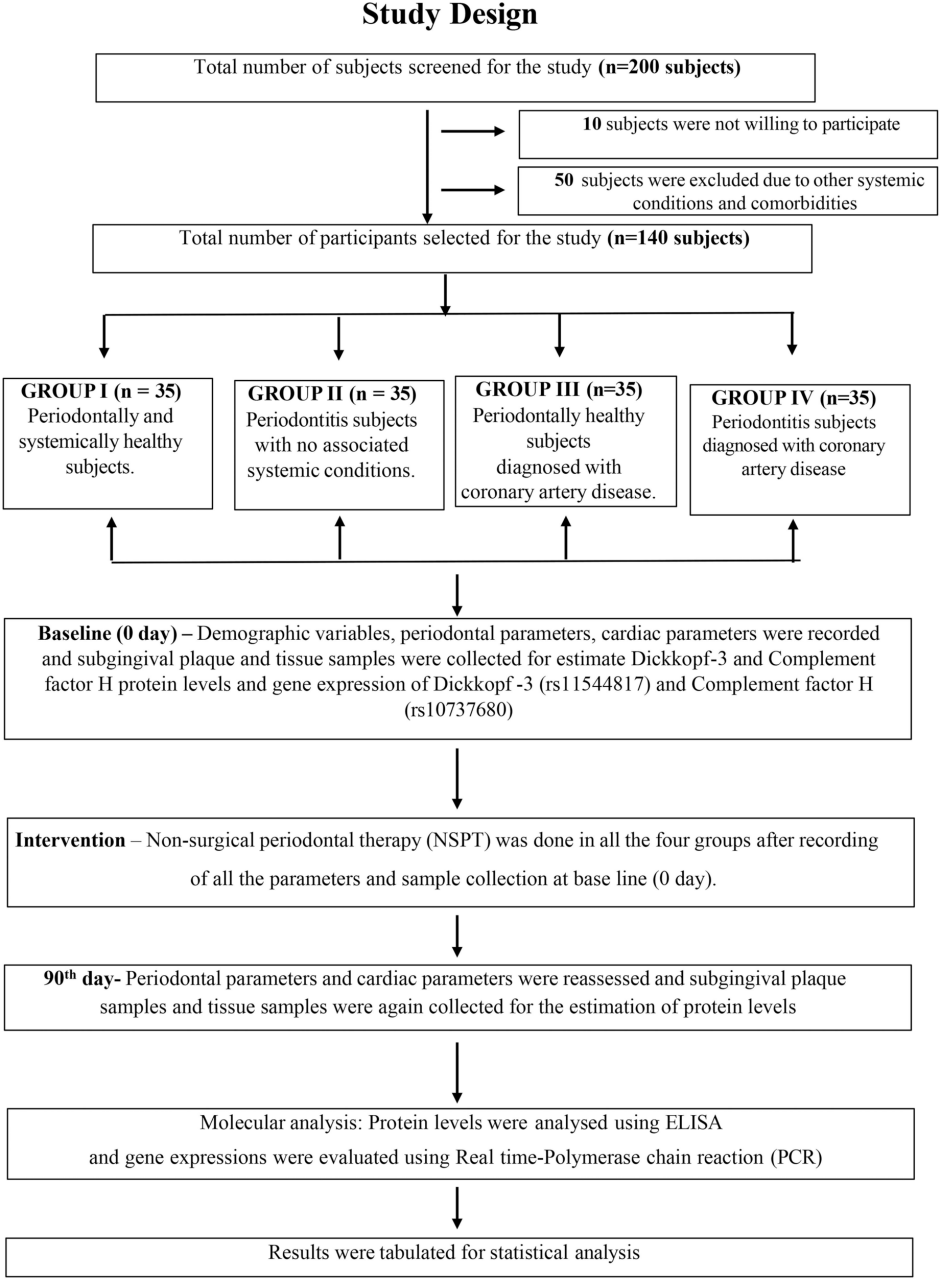
Study design flowchart.

A total of 200 subjects were screened from the Department of Periodontics, Meenakshi Ammal Dental College and Hospital, and the Department of Cardiology, Kilpauk Medical College and Hospital, Chennai. Of the recruited participants, 10 were unwilling to participate and 50 were excluded due to systemic conditions and comorbidities based on inclusion and exclusion criteria. Finally, 140 participants were selected and were divided into four groups as follows: H group, 35 periodontally and systemically healthy individuals; P group, 35 patients with periodontitis and no associated systemic diseases; CAD group, 35 periodontally healthy individuals diagnosed with CAD; and P + CAD group, 35 patients with periodontitis who were diagnosed with CAD (Figure 1).

### 2.2 Sample size

The required sample size for this study was determined based on the prevalence of periodontal disease and CAD in the region, as documented in hospital records and supported by evidence from previous studies. A power analysis indicated that a minimum of 120 subjects (30 participants per group) would achieve a statistical power of 95%. To account for potential dropouts, the sample size was increased to 140 participants, with 35 subjects allocated to each group. This approach ensured that the study maintained sufficient power to detect clinically meaningful differences between the groups, thereby upholding methodological rigor.

### 2.3 Parameters assessed

The study assessed demographic variables, namely, age, height, weight, BMI, socioeconomic status, lifestyle, and diet, at baseline. The periodontal parameters included plaque index (PI), BOP, probing pocket depth (PPD), and CAL (12, 13). BOP was calculated as the percentage of bleeding sites per tooth, while PPD and CAL were measured at six sites per tooth. These parameters were recorded at baseline and day 90 using a Williams Periodontal Probe by two calibrated examiners (JM and BM), with satisfactory inter- and intra-examiner reliability (*κ* = 0.70 and 0.69). Cardiac parameters, such as total cholesterol (TC), high-density lipoprotein (HDL-c), low-density lipoprotein (LDL-c), triglyceride levels (TG), and systolic and diastolic blood pressure, were obtained from the hospital records both at baseline and on day 90.

### 2.4 Collection of subgingival plaque and tissue samples

To minimize sample contamination, the participants rinsed their mouths with distilled water before sample collection. Subgingival plaque and gingival tissue samples were obtained using sterile manual universal curettes (Hu-Friedy, Chicago, IL, USA) under standard aseptic conditions. In the periodontitis groups (Groups P and P + CAD), plaque samples were harvested from the sites with the deepest periodontal pockets. In the periodontally healthy groups (Groups H and CAD), gingival tissue samples were collected from sites indicated for therapeutic procedures, such as crown lengthening or intentional extractions, where clinically healthy gingival tissue could be ethically obtained.

The samples from the four study groups were immediately placed in sterilized vials containing 95% absolute ethanol and stored at −80°C for subsequent analysis of DKK-3 (rs11544817) and CFH (rs10737680) gene expression and protein levels. Following sample collection, all the participants underwent NSPT and received personalized oral hygiene instructions. At the 90-day follow-up, the periodontal and cardiac parameters were reassessed and subgingival plaque and gingival tissue samples were collected again from the periodontitis and periodontally healthy groups, respectively. The expression levels of DKK-3 and CFH were then analyzed and compared between the groups.

### 2.5 Molecular analysis

The molecular analysis was conducted in the Central Research Laboratory, Meenakshi Ammal Dental College and Hospital, Chennai. After the collection of the subgingival plaque and tissue samples from the four groups, molecular analyses were performed to detect the protein levels of DKK-3 and CFH using enzyme-linked immunosorbent assay (ELISA). The gene expression of DKK-3 (rs11544817) and CFH (rs10737680) was analyzed using real-time polymerase chain reaction (PCR). The molecular analysis was conducted using a commercially available ELISA kit (Elabscience, Houston, TX, USA) and the double antibody sandwich technique, following the manufacturer's instructions. After bringing all the reagents and subgingival samples to room temperature and removing the ethanol, 1% phosphate-buffered saline (PBS) was added. Samples, standards, and blanks (100 µL each) were loaded into wells and incubated at 37°C for 90 min. After sequential steps of incubation with biotinylated detection antibody, horseradish peroxidase (HRP) conjugate, and substrate reagent, the reaction was stopped using a stop solution (50 µL), resulting in a color change from blue to yellow. The optical density was measured at 450 nm using an ELISA plate reader. The assay's standard curve for DKK-3 ranged from 2.724 to 1,000 ng/mL, with a sensitivity of 0.242 ng/mL, while for CFH, it ranged from 2.874 to 1,000 ng/mL, with a sensitivity of 0.216 ng/mL.

DNA (100 µL) was isolated from the subgingival tissue samples using the Xpress DNA kit (MagGenome Technologies Pvt. Ltd., Cochin, India), following the manufacturer's protocol. DNA quantification was performed using a NanoDrop spectrophotometer (Thermo Fisher Scientific, Wilmington, DE, USA) and real-time PCR was conducted using the Rotor-Gene Q 6000 thermal cycler (Qiagen, Hilden, Germany). Specific primers were used to determine the gene expression of DKK-3 (rs11544817) (forward primer: AACAGTGGTTTTTGCATTGC; and reverse primer: TTGGCATTGCTCATCTGCG) and CFH (rs10737680) (forward primer: CATAGCCCCTTCTCAGAACAG; and reverse primer: GTAAAGGATCTCATGATACTGG) (PrimeX, Gujarat, India). The 20 µL reaction mix included 10 µL of PCR master mix (Ampliqon, Denmark), 2 µL each of the forward and reverse primers, 2.5 µL of DNA, and 3.5 µL of distilled water. The real-time PCR conditions were as follows: initial denaturation at 95°C for 5 min; 40 cycles of denaturation at 95°C for 30 s; annealing at 55°C for 30 s; extension at 72°C for 30 s; followed by final extension at 72°C.

A total of 1 g of agarose was dissolved in 50 mL of a Tris-acetate ethylenediaminetetraacetic acid (EDTA) 1X (TAE) buffer and heated. Then, the agarose gel was cast with 5 L of ethidium bromide. The solution was then poured into the gel cassettes and allowed to cool and solidify. The gel cassette was then placed in the tank containing the 50X TAE buffer. A total of 5 µL of the real-time PCR product was then loaded into each well with a 100–3,000 bp DNA ladder to track the molecular weight of the PCR product. Electrophoresis was then carried out at 150 mA and the gels were visualized using a Medccare Gel Documentation System (Medccare Pvt. Ltd., India).

### 2.6 Statistical analysis

Statistical analysis was done using the Statistical Package for the Social Sciences (SPSS) version 26.0 (IBM Corp, Armonk, NY, USA). Inferential statistics were conducted using one-way ANOVA and followed by a *post-hoc test* to assess the pairwise comparison. To compare the proportions between the groups, the chi-square test was used. To assess the difference between baseline (day 0) and day 90 following scaling and root planing, the independent sample *t*-test and unpaired *t*-test were used. The correlation analysis was conducted using Pearson’s correlation to assess the correlation between the determinants. In the present study, a *p*-value <0.05 was considered the level of significance and *p* <0.001 was considered to be highly statistically significant. A multiple linear regression analysis was conducted to determine the factors that influence DKK-3 (rs11544817) and CFH (rs10737680). A univariate logistic regression analysis was conducted to determine the risk factors for the periodontitis and CAD group.

## 3 Results

When comparing the demographic variables between the groups, the mean age was the oldest in the P + CAD group (51.57 ± 5.19 years), showing a highly significant difference (*p* < 0.001). The mean weight and BMI were higher in the P + CAD group, with statistically significant differences (*p*-values < 0.001). Mean monthly income was lowest in the P + CAD group with Rs. 11,828.57 ± 2,572.32, which was statistically significant (*p* < 0.001). The other demographic parameters, namely, height, lifestyle habits (active vs. sedentary), and dietary habits (vegetarian vs. non-vegetarian), were non-significant among the groups (Table 1).

**Table 1:** Intergroup and intragroup comparisons of the demographic variables, periodontal parameters, cardiac parameters, protein levels of DKK-3 and CFH, and gene expression of DKK-3 (rs11544817) and CFH (rs10737680) among the four groups at baseline (day 0) and day 90.

Variable	Group	Mean ± std. deviation		*p*-value (intragroup)
		Day 0	Day 90	
Age (years)	H	39.2 ± 5.95	—	—
	P	49.23 ± 5.31	—	
	CAD	46 ± 4.38	—	
	P + CAD	51.57 ± 5.19	-	
	*p*-value (intergroup)	<0.001**	—	
Height (cm)	H	165.71 ± 10.58	—	—
	P	165.09 ± 7.28	—	
	CAD	167.88 ± 6.45	—	
	P + CAD	164.29 ± 7.7	—	
	*p*-value (intergroup)	0.294^NS^	—	
Weight (kg)	H	59.89 ± 9.14	—	—
	P	63.88 ± 8.23	—	
	CAD	73.23 ± 12.41	—	
	P + CAD	74.51 ± 9.26	—	
	*p*-value (intergroup)	<0.001**	—	
BMI (kg/m^2^)	H	21.61 ± 2.07	—	—
	P	23.27 ± 2.21	—	
	CAD	26.38 ± 2.67	—	
	P + CAD	27.18 ± 4.61	—	
	*p*-value (intergroup)	<0.001**	—	
Monthly income (Rs)	H	16,400 ± 2,724.62	—	—
	P	14,642.86 ± 3,052.41	—	
	CAD	15,085.71 ± 4,421.68	—	
	P + CAD	11,828.57 ± 2,572.32	—	
	*p*-value (intergroup)	<0.001**	—	
Lifestyle (active)	H	28 (80%)	—	—
	P	22 (62.9%)	—	
	CAD	25 (71.4%)	—	
	P + CAD	18 (51.4%)	—	
	*p*-value (intergroup)	0.472^NS^	—	
Lifestyle (sedentary)	H	7 (20%)	—	—
	P	13 (37.1%)	—	
	CAD	10 (28.6%)	—	
	P + CAD	17 (48.6%)	—	
	*p*-value (intergroup)	0.472^NS^	—	
Diet (vegetarian)	H	4 (11.4%)	—	0.382^NS^
	P	4 (11.4%)	—	
	CAD	6 (17.1%)	—	
	P + CAD	5 (14.3%)	—	
	*p*-value (intergroup)	0.382^NS^	—	
Diet (non-vegetarian)	H	31 (88.6%)	—	0.382^NS^
	P	31 (88.6%)	—	
	CAD	29 (82.9%)	—	
	P + CAD	30 (85.7%)	—	
	*p*-value (intergroup)	0.382^NS^	—	
Plaque index	H	1.15 ± 0.19	0.93 ± 0.19	<0.001**
	P	2.52 ± 0.25	1.79 ± 0.29	<0.001**
	CAD	1.29 ± 0.25	0.97 ± 0.21	<0.001**
	P + CAD	2.6 ± 0.25	1.53 ± 0.53	<0.001**
	*p*-value (intergroup)	<0.001**	<0.001**	-
Bleeding on probing (%)	H	19.17 ± 6.05	15.76 ± 3.92	<0.001**
	P	71.38 ± 11.27	68.52 ± 11.41	<0.001**
	CAD	31.46 ± 33.59	22.16 ± 2.39	<0.001**
	P + CAD	78.03 ± 7.79	73.48 ± 9.21	<0.001**
	*p*-value (intergroup)	<0.001**	<0.001**	<0.001**
Probing pocket depth (mm)	H	1.74 ± 0.41	1.39 ± 0.37	<0.001**
	P	5.1 ± 0.41	3.62 ± 0.82	<0.001**
	CAD	2.27 ± 0.43	1.71 ± 0.36	<0.001**
	P + CAD	5.25 ± 0.67	3.2 ± 0.86	<0.001**
	*p*-value (intergroup)	<0.001**	<0.001**	<0.001**
Clinical attachment level (mm)	H	1.74 ± 0.41	1.39 ± 0.37	<0.001**
	P	5.13 ± 0.42	3.63 ± 0.83	<0.001**
	CAD	2.27 ± 0.43	1.71 ± 0.36	<0.001**
	P + CAD	5.28 ± 0.67	3.22 ± 0.86	<0.001**
	*p*-value (intergroup)	<0.001**	<0.001**	—
Total cholesterol (mg/dL)	H	156.11 ± 8.2	155.23 ± 7.56	0.027*
	P	161.03 ± 12.31	160.77 ± 11.98	0.045*
	CAD	218.49 ± 22.17	215.4 ± 21.61	<0.001**
	P + CAD	220.94 ± 18.23	215.86 ± 17.51	<0.001**
	*p*-value (intergroup)	<0.001**	<0.001**	—
High-density lipoprotein (m g/dL)	H	75.23 ± 7.55	75.89 ± 7.92	0.013*
	P	74.23 ± 6.68	76.11 ± 6.68	0.027*
	CAD	43.74 ± 3.65	47.74 ± 3.36	<0.001**
	P + CAD	39.66 ± 3.72	45.8 ± 3.45	<0.001**
	*p*-value (intergroup)	<0.001**	<0.001**	—
Low-density lipoprotein (mg/dL)	H	76.74 ± 5.89	77.94 ± 5.99	0.043*
	P	85.06 ± 4.61	84.23 ± 4.51	0.018*
	CAD	125.11 ± 6.4	113.31 ± 6.52	<0.001**
	P + CAD	126.74 ± 4.69	114.63 ± 6.3	<0.001**
	*p*-value (intergroup)	<0.001**	<0.001**	
Triglycerides (mg/dL)	H	132.29 ± 6.57	130.69 ± 6.41	0.033*
	P	143.2 ± 6.02	142.2 ± 6.2	0.059*
	CAD	193.14 ± 8.3	173.86 ± 8.56	<0.001**
	P + CAD	195.29 ± 5.78	174.4 ± 5.83	<0.001**
	*p*-value (intergroup)	<0.001**	<0.001**	—
Systolic blood pressure (mmHg)	H	120 ± 5.42	119.43 ± 4.82	0.571^NS^
	P	120.29 ± 7.47	120 ± 5.42	0.838^NS^
	CAD	123.14 ± 6.76	120.86 ± 5.07	0.118^NS^
	P + CAD	123.14 ± 7.18	120.31 ± 19.87	0.416^NS^
	*p*-value (intergroup)	0.079	0.095	—
Diastolic blood pressure (mmHg)	H	77.71 ± 4.9	78.86 ± 4.04	0.292^NS^
	P	80 ± 5.42	81.14 ± 5.3	0.353^NS^
	CAD	76 ± 5.53	79.71 ± 5.68	0.201^NS^
	P + CAD	80 ± 6.42	83.71 ± 5.47	0.364^NS^
	*p*-value (intergroup)	0.072	0.081	—
DKK-3 (ng/mL)	H	1.1 ± 0.22	0.6 ± 0.19	<0.01*
	P	1.39 ± 0.16	0.97 ± 0.23	<0.001**
	CAD	1.68 ± 0.17	1.16 ± 0.11	<0.001**
	P + CAD	1.87 ± 0.35	1.5 ± 0.15	<0.001**
	*p*-value (intergroup)	<0.001**	<0.001**	—
CFH (ng/mL)	H	29.83 ± 3.21	40.6 ± 11.73	<0.01*
	P	17.41 ± 2.38	28.77 ± 4.84	<0.001**
	CAD	12.49 ± 1.92	19.67 ± 2.97	<0.001**
	P + CAD	4.87 ± 1.94	13.54 ± 2.73	<0.001**
	*p*-value (intergroup)	<0.001**	<0.001**	—
DKK-3 (rs11544817) (%)	H	28.35 ± 1.23	—	—
	P	25.41 ± 3.34	—	—
	CAD	22.82 ± 1.6	—	—
	P + CAD	21.05 ± 1.39	—	—
	*p*-value (intergroup)	<0.001**	—	—
CFH (rs10737680) (%)	H	5.17 ± 0.98	—	—
	P	8.28 ± 0.76	—	—
	CAD	10.66 ± 1.17	—	—
	P + CAD	16.16 ± 1.68	—	—
	*p*-value (intergroup)	<0.001**		

NS, non-significant; H, healthy subjects; P, periodontitis; CAD, coronary artery disease; DKK-3, Dickkopf-3; CFH, complement factor H.

Data were expressed as mean ± standard deviation (SD). Intergroup comparisons among the study groups were performed using one-way analysis of variance (ANOVA) followed by Tukey's *post-hoc* test. Intragroup (baseline vs. 90-day) comparisons were conducted using the paired Student's *t*-test. The statistical analyses were conducted using SPSS v26 (IBM Corp., USA), with a significance level of *p* < 0.05 and a highly significant level of *p* < 0.05.

* *p* < 0.05; **p < 0.001.

At baseline and 90 days post-NSPT, all the groups showed significant improvements in PI, BOP, PPD, and CAL (intragroup and intergroup comparisons, *p* < 0.001). Similarly, TC, HDL-c, LDL-c, and TG levels demonstrated significant differences within and between groups (*p* < 0.001), with highly significant changes within the CAD and P + CAD groups and significant differences between the healthy and periodontitis groups (*p* < 0.05). Systolic and diastolic blood pressure remained unchanged across the groups (*p* > 0.05), indicating that NSPT had minimal influence on this variable (Table 1).

In the intragroup and intergroup comparisons, the mean DKK-3 protein levels significantly decreased from baseline to day 90 post-NSPT in all the groups. Conversely, CFH protein levels significantly increased in all groups, showing statistical significance (*p* < 0.001) (Table 1). Regarding gene expression, DKK-3 (rs11544817) and CFH (rs10737680) were assessed based on comparative quantification cycle (Cq) values, wherein higher percentage values corresponded to lower gene expression and lower values indicated higher expression.

For DKK-3, the healthy subjects showed the highest percentage (28.35% ± 1.23%), reflecting the lowest gene expression, while the lowest percentage (21.05% ± 1.39%) was observed in the P + CAD group, indicating the highest DKK-3 expression. The significant decreasing trend in percentage values from the healthy group to the P + CAD group (*p* < 0.001) suggests an upregulation of DKK-3 expression in the presence of both periodontitis and cardiovascular disease. Conversely, for CFH, the healthy participants exhibited the lowest percentage (5.17% ± 0.98%), corresponding to the highest gene expression, whereas the P + CAD group showed the highest percentage (16.16% ± 1.68%), indicating the lowest expression. This progressive increase in percentage values across the groups (*p* < 0.001) reflects a significant downregulation of CFH expression with advancing disease severity (Table 1).

The periodontal parameters, namely, PI, BOP, PPD, and CAL, were all significantly higher in the P + CAD group vs. the H group, the P + CAD group vs. the CAD group, and the P + CAD group vs. the P group (*p* < 0.001). The differences in these parameters were also statistically significant at baseline and day 90 (*p* < 0.01). At baseline and day 90, there were significantly higher levels of TC, HDL-c, LDL-c, and TG in the P + CAD group vs. the H group and the P + CAD group vs. the P group (*p* < 0.001), while the P + CAD group vs. the CAD group comparison showed significant differences (*p* < 0.05). However, no significant differences were noted in systolic or diastolic blood pressure among these groups (*p* > 0.05). At baseline and day 90, the mean levels of DKK-3 (ng/mL) and CFH (ng/mL) in the P + CAD group vs. the H group and the P + CAD group vs. the P group were significantly higher (*p* < 0.001), while these variables were significantly different in the P + CAD group vs. CAD group comparison (*p* = 0.007). Similarly, CFH levels showed highly significant differences between the P + CAD group vs. all other groups (*p* < 0.001). In addition, significant differences in the percentage distribution of the DKK-3 (rs11544817) and CFH (rs10737680) genotypes were observed at baseline between the P + CAD group and the other groups, with the DKK-3 differences being highly significant in the P + CAD group vs. H group and P + CAD group vs. P group comparisons (*p* < 0.001) and significant in the P + CAD group vs. CAD group comparison (*p* = 0.003), while the CFH differences were highly significant across all comparisons (*p* < 0.001) (Table 2).

**Table 2:** Multiple comparisons of the mean difference and test of significance of the demographic variables, periodontal parameters, cardiac parameters, protein levels of DKK-3 and CFH, and gene expression of DKK-3 (rs11544817) and CFH (rs10737680) among all the groups at baseline (day 0) and day 90.

Variable	Groups	Mean difference	*p*-value	Mean difference	*p*-value
		Day 0		Day 90	
Plaque index	P + CAD group vs. H group	1.45	<0.001**	0.6	<0.001**
	P + CAD group vs. P group	0.08	0.004*	−0.26	0.007*
	P + CAD group vs. CAD group	1.31	<0.001**	0.55	<0.001**
Bleeding on probing (%)	P + CAD group vs. H group	58.86	<0.001**	57.73	<0.001**
	P + CAD group vs. P group	6.65	0.007*	4.96	0.047*
	P + CAD group vs. CAD group	46.57	<0.001**	51.32	<0.001**
Probing pocket depth (mm)	P + CAD group vs. H group	3.51	<0.001**	1.81	<0.001**
	P + CAD group vs. P group	0.15	0.009*	−0.43	0.04*
	P + CAD group vs. CAD group	2.98	<0.001**	1.49	<0.001**
Clinical attachment level (mm)	P + CAD group vs. H group	3.54	<0.001**	1.84	<0.001**
	P + CAD group vs. P group	0.15	0.007*	−0.41	0.042*
	P + CAD group vs. CAD group	3.01	<0.001**	1.51	<0.001**
Total cholesterol (mg/dL)	P + CAD group vs. H group	64.83	<0.001**	60.629	<0.001**
	P + CAD group vs. P group	59.91	<0.001**	55.086	<0.001**
	P + CAD group vs. CAD group	12.46	0.037*	10.457	0.037*
High-density lipoprotein (mg/dL)	P + CAD group vs. H group	−35.57	<0.001**	−41.086	<0.001**
	P + CAD group vs. P group	−34.57	<0.001**	−30.314	<0.001**
	P + CAD group vs. CAD group	−14.09	0.049*	−11.943	0.042*
Low-density lipoprotein (mg/dL)	P + CAD group vs. H group	48.8	<0.001**	37.886	<0.001**
	P + CAD group vs. P group	41.69	<0.001**	30.4	<0.001**
	P + CAD group vs. CAD group	11.63	0.027*	11.314	0.046*
Triglycerides (mg/dL)	P + CAD group vs. H group	63	<0.001**	43.714	<0.001**
	P + CAD group vs. P group	52.09	<0.001**	32.2	<0.001**
	P + CAD group vs. CAD group	12.14	0.036*	10.543	0.023*
Systolic blood pressure (mmHg)	P + CAD group vs. H group	3.143	0.322^NS^	0.886	0.997^NS^
	P + CAD group vs. P group	2.857	0.474^NS^	0.314	0.967^NS^
	P + CAD group vs. CAD group	0	0.997^NS^	−0.543	0.954^NS^
Diastolic blood pressure (mmHg)	P + CAD group vs. H group	2.286	0.538^NS^	4.857	0.07^NS^
	P + CAD group vs. P group	0	1^NS^	2.571	0.234^NS^
	P + CAD group vs. CAD group	4	0.21^NS^	4	0.09^NS^
DKK-3 (ng/mL)	P + CAD group vs. H group	0.19	<0.001**	0.9	<0.001**
	P + CAD group vs. P group	0.48	<0.001**	0.33	<0.001**
	P + CAD group vs. CAD group	0.77	0.007*	0.53	<0.001**
CFH (ng/mL)	P + CAD group vs. H group	−24.96	<0.001**	−27.06	<0.001**
	P + CAD group vs. P group	−12.54	<0.001**	−15.23	<0.001**
	P + CAD group vs. CAD group	−7.62	<0.001**	−6.13	0.001*
DKK-3 (rs11544817) (%)	P + CAD group vs. H group	−7.306	<0.001**	—	—
	P + CAD group vs. P group	−4.361	<0.001**	—	—
	P + CAD group vs. CAD group	−1.771	0.003*	—	—
CFH (rs10737680) (%)	P + CAD group vs. H group	5.493	<0.001**	—	—
	P + CAD group vs. P group	7.878	<0.001**	—	—
	P + CAD group vs. CAD group	10.983	<0.001**	—	—

NS, non-significant; H, healthy subjects; P, periodontitis; CAD, coronary artery disease; DKK-3, Dickkopf-3; CFH, complement factor H.

Data are shown as mean ± SD and mean difference (95% CI). One-way ANOVA with Bonferroni *post-hoc* multiple comparisons was used to analyze the differences among the groups. The statistical analyses were conducted using SPSS v26 (IBM Corp., USA) with a significance level of *p* < 0.05 and a highly significant level of *p* < 0.05.

* *p* < 0.05; **p< 0.001.

In the correlation analysis of DKK-3 and CFH between baseline and day 90, DKK-3 and CFH showed highly significant correlations (*p* < 0.001) for PI, BOP, PPD, CAL, TC, HDL-c, LDL-c, and TG and no significant correlation was found for systolic or diastolic blood pressure (Table 3).

**Table 3:** Correlation between the variables overall and DKK-3 and CFH at baseline (day 0) and day 90.

Variable	Correlation/*p*-value	DKK-3 (ng/mL)	CFH (ng/mL)	DKK-3 (ng/mL)	CFH (ng/mL)
		Day 0		Day 90	
Plaque index	Correlation	0.337	−0.567	0.317	−0.207
	*p*-value	<0.001**	<0.001**	<0.001**	0.001**
Bleeding on probing (%)	Correlation	0.377	−0.612	0.532	−0.473
	*p*-value	<0.001**	<0.001**	<0.001**	<0.001**
Probing pocket depth (mm)	Correlation	0.37	−0.603	0.403	−0.326
	*p*-value	<0.001**	<0.001**	<0.001**	<0.001**
Clinical attachment level (mm)	Correlation	0.369	−0.603	0.405	−0.329
	*p*-value	<0.001**	<0.001**	<0.001**	<0.001**
Total cholesterol (mg/dL)	Correlation	0.636	−0.726	0.676	−0.723
	*p*-value	<0.001**	<0.001**	<0.001**	<0.001**
High-density lipoprotein (mg/dL)	Correlation	−0.693	0.792	−0.737	0.763
	*p*-value	<0.001**	<0.001**	<0.001**	<0.001**
Low-density lipoprotein (mg/dL)	Correlation	0.728	−0.828	0.759	−0.788
	*p*-value	<0.001**	<0.001**	<0.001**	<0.001**
Triglycerides (mg/dL)	Correlation	0.699	−0.843	0.757	−0.81
	*p*-value	<0.001**	<0.001**	<0.001**	<0.001**
Systolic blood pressure (mmHg)	Correlation	0.069	−0.174	0.053	−0.033
	*p*-value	0.185^NS^	−0.645^NS^	0.534^NS^	0.699^NS^
Diastolic blood pressure (mg/dL)	Correlation	0.089	−0.055	0.192	−0.127
	*p*-value	0.296^NS^	0.518^NS^	0.085^NS^	0.107^NS^

NS, non-significant; H, healthy subjects; P, periodontitis; CAD, coronary artery disease; DKK-3, Dickkopf-3; CFH, complement factor H.

The correlations between continuous variables were assessed using Pearson's correlation test. The correlation coefficients (*r*) and *p-*values are shown. All the analyses were performed in SPSS v26 (IBM Corp., USA), with *p* < 0.05 indicating statistical significance.

** *p*< 0.001.

The multiple linear regression revealed that elevated DKK-3 protein levels were linked to increased DKK-3 (rs11544817) expression and decreased CFH (rs10737680) expression, indicating a higher risk for periodontitis and CAD. Conversely, lower CFH protein levels also showed a strong association with elevated levels of the other study variables, reinforcing their contribution to elevated risk (Table 4).

**Table 4:** Multiple linear regression coefficients at baseline (0 day).

Dependent variable	Independent variable	Coefficient	Std. error	95% confidence interval		*p*-value
				Lower limit	Upper limit	
DKK-3 (ng/mL)	DKK-3 (rs11544817) (%)	−0.027	0.009	−0.045	−0.009	0.004*
	CFH (rs10737680) (%)	0.049	0.007	0.034	0.064	0.001**
CFH (ng/mL)	DKK-3 (rs11544817) (%)	0.698	0.142	0.417	0.978	0.001**
	CFH (rs10737680) (%)	−1.584	0.116	−1.814	−1.354	0.001**

DKK-3, Dickkopf-3; CFH, complement factor H.

Multiple linear regression was performed using the ordinary least squares method to assess the relationship between protein concentrations and gene expression levels of DKK-3 and CFH. The dependent variables were DKK-3 (ng/mL) and CFH (ng/mL), and the independent variables were DKK-3 (rs11544817) (%) and CFH (rs10737680) (%). The significance of each predictor was tested using Student's *t*-test for regression coefficients, and the overall model fit was evaluated using the *F*-test derived from the ANOVA results of the regression analysis. Statistical analyses were performed using SPSS v26 (IBM Corp., USA); *p* < 0.05 was considered statistically significant, and *p* ≤ 0.001 was considered highly significant.

** *p* ≤ 0.001.

The univariate logistic regression identified older age, male sex, sedentary lifestyle, a non-vegetarian diet, and obesity as significant risk factors for periodontitis and CAD. Strong associations were observed in the comparison of the periodontal indices (PI, BOP, PPD, and CAL) and lipid markers (TC, LDL-c, and TG), while HDL-c was protective. DKK-3 (rs11544817) was a major risk factor, while CFH (rs10737680) had a protective role (Table 5).

**Table 5:** Univariate logistic regression analysis of the study variables in the P + CAD group at baseline (day 0).

Independent variable	Odds ratio (95% CI)	*p*-value
Age (year)	1.341 (1.21–1.486)	<0.001**
Sex: male	1.509 (0.632–3.606)	0.354^NS^
Lifestyle: sedentary	2.462 (0.984–6.159)	0.054^NS^
Diet: non-vegetarian	1.292 (0.398–4.187)	0.67^NS^
Height (cm)	1.001 (0.955–1.049)	0.982^NS^
Weight (kg)	1.103 (1.054–1.154)	<0.001**
BMI (kg/m^2^)	1.477 (1.264–1.725)	<0.001**
Plaque index	46.455 (8.511–65.576)	<0.001**
Bleeding on probing (%)	1.392 (1.174–1.651)	<0.001**
Probing pocket depth (mm)	15.291 (4.456–52.472)	<0.001**
Clinical attachment level (mm)	15.276 (4.445–52.49)	<0.001**
Total cholesterol (mg/dL)	1.083 (1.047–1.12)	<0.001**
High-density lipoprotein (mg/dL)	0.901 (0.866–0.937)	<0.001**
Low-density lipoprotein (mg/dL)	1.257 (1.129–1.4)	<0.001**
Triglyceride (mg/dL)	1.265 (1.143–1.401)	<0.001**
Systolic blood pressure (mmHg)	1.048 (0.99–1.108)	0.104^NS^
Diastolic blood pressure (mmHg)	1.029 (0.963–1.1)	0.398^NS^
DKK-3 (ng/mL)	38.134 (6.334–58.464)	<0.001**
CFH (ng/mL)	0.192 (0.032–1.162)	0.072^NS^
DKK-3 (rs11544817) (%)	0.476 (0.368–0.615)	<0.001**
CFH (rs10737680) (%)	20.731 (3.951–48.79)	0.053^NS^

NS, non-significant; DKK-3, Dickkopf-3; CFH, complement factor H.

A univariate logistic regression analysis was used to assess the statistical significance of each predictor and the odds ratio (OR) with the 95% confidence interval (CI) was reported. The analyses were conducted using SPSS version 26 (IBM Corp., USA), with a significance level set at *p* < 0.05 and a highly significant level of *p* < 0.001. The dependent variables were the presence of periodontitis and coronary artery disease [P + CAD]. The independent variables entered individually into the model included age, sex, height, weight, BMI, monthly income, lifestyle, PI, gingival index (GI), PPD, CAL, total cholesterol, triglycerides, HDL-c, LDL-c, DKK-3 protein (ng/mL), CFH protein (ng/mL), DKK-3 gene expression (%), and CFH gene expression (%).

** *p* < 0.001.

## 4 Discussion

Periodontitis is an inflammatory disease caused by a microbial imbalance, leading to the destruction of tooth-supporting tissues. It is strongly linked to various systemic conditions, including CAD (1). The mechanisms behind this connection involve oral microbial load, systemic inflammation, altered hemostasis, and increased oxidative stress. Periodontal pathogens found in atherosclerotic plaque suggest their role in accelerating atherosclerosis, making chronic, untreated periodontitis a significant risk factor for cardiovascular disease (2).

CAD is a leading cause of mortality in India, with strong evidence linking it to periodontitis. The two conditions share similar pathological mechanisms, making early detection of periodontal infections essential to reduce the risk of CAD. Biomarkers from body fluids such as saliva, serum, GCF, and other oral samples provide non-invasive indicators of disease severity. Among these, subgingival samples offer unique advantages, as they are directly associated with periodontal pathogens and disease progression. They contain valuable biomarkers that help in understanding both oral and systemic health, aiding early diagnosis and targeted treatment for periodontal and cardiovascular diseases (3).

Gene expression of pro-inflammatory biomarkers, such as cytokines and matrix metalloproteinases, is elevated in periodontal disease and linked to systemic conditions, such as cardiovascular disease (4). This study analyzed the DKK-3 and CFH protein levels in the subgingival plaque and gene expression in the subgingival tissue of patients with periodontitis and CAD. DKK-3 and CFH are key biomarkers in inflammation, but their role in both diseases remains unclear. In this research, we hypothesized that NSPT may modulate DKK-3 and CFH protein levels in these patients. The P + CAD group had the oldest age and the highest weight and BMI, correlating with greater CAD and periodontitis risk, as aging, obesity, and inflammation are major contributing factors (14, 15). Lower socioeconomic status further increases susceptibility due to limited healthcare access and poor oral hygiene (16–18) (Table 1).

The measurement of the periodontal parameters at baseline showed that the P + CAD group had significantly higher PI, BOP, PPD, and CAL values compared to the other groups, aligning with Mahendra et al. and Bilgin Çetin et al. (14, 19). These parameters indicate the severity of periodontal disease in individuals with periodontitis and CAD. After NSPT, all the groups showed a significant reduction in these parameters, with the P + CAD group showing the greatest improvement, which is consistent with the studies by Al-Isa et al. and Goswamy et al. (20, 21). The measurement of the cardiac parameters at baseline revealed elevated LDL-c, TC, and TG, as well as decreased HDL-c, in the P + CAD group, supporting findings by Fentoglu et al. and Mahendra et al., who linked hyperlipidemia to CAD and periodontal disease (22, 23). After NSPT, the lipid profiles improved significantly, particularly in the P + CAD group, as a result of reduced inflammation, consistent with Sania et al. (Tables 1, 2) (24).

The protein levels of DKK-3 and CFH were also assessed. DKK-3 levels were highest in the P + CAD group and significantly decreased after NSPT, reflecting its role in inflammation, as suggested by Cheng et al. and Xu et al. (25, 26). CFH levels were highest in the healthy group and lowest in the P + CAD group, suggesting its anti-inflammatory role, in line with Kiss et al. (27). Both DKK-3 and CFH were positively correlated with the lipid and periodontal parameters. The gene expression analysis revealed a higher expression of DKK-3 in the P + CAD group, consistent with Piek et al. and a lower expression of CFH, in line with Nielsen et al. and Salminen et al., indicating potential genetic links to periodontitis and CAD (28–30). These findings emphasize the importance of DKK-3 and CFH in the pathogenesis of both diseases. Significant findings were obtained when comparing DKK-3 and CFH proteins at baseline and at day 90, suggesting that higher DKK-3 levels and lower CFH protein levels are correlated with the severity of periodontitis and higher lipid profiles (Tables 1–3).

The multiple linear regression revealed that elevated DKK-3 protein levels were associated with increased DKK-3 (rs11544817) expression and decreased CFH (rs10737680) expression, indicating a higher risk for periodontitis and CAD. Conversely, lower CFH protein levels also showed similar associations, emphasizing their role in increasing risk. The univariate logistic regression analysis found significant odds ratios for periodontitis and CAD, underscoring the impact of the key study variables.

This study is unique in evaluating the odds ratios to establish a strong link between these variables and coronary events (Tables 4, 5).

To the best of our knowledge, this is the first study to evaluate the protein levels and gene expression of DKK-3 (rs11544817) and CFH (rs10737680) in subgingival plaque and tissue samples in patients with periodontitis with or without CAD and in healthy controls. Overall, this study explored the relationship between periodontitis and CAD, revealing a new link. It highlights the importance of NSPT in reducing inflammation and consequently reducing the risk of subsequent coronary events and suggests the potential use of these proteins as diagnostic biomarkers for early CAD risk detection and to evaluate treatment outcomes for improved patient care.

While existing studies have examined biomarkers and subgingival microbiome profiles in periodontal diseases, none have investigated the gene expression or genetic variation of DKK-3 (rs11544817) and CFH (rs10737680) in subgingival tissue samples to explore the genetic link between periodontitis and CAD. This novel study sought to address this gap by evaluating the gene expression of Dickkopf-3 (rs11544817), providing new insights into its pathophysiological relevance and diagnostic potential. Moreover, this study can serve as a foundation for future research on genetic mutations related to inflammatory diseases, particularly those exploring the association between periodontitis and CAD.

However, the current study has certain limitations that warrant consideration. First, its cross-sectional design restricted the establishment of causal relationships and the lack of long-term follow-up limited its ability to observe the evolution of conditions over time. In addition, the study did not assess the expression of DKK-3 and CFH in serum samples to capture the comprehensive inflammatory load.

## 5 Conclusion

This study provides the first evidence of DKK-3 and CFH protein expression in subgingival plaque and tissue samples from patients with periodontitis with or without CAD compared to healthy controls. The significant reduction in DKK-3 levels and the increase in CFH levels following NSPT suggest their potential roles as pro- and anti-inflammatory markers, respectively. In addition, the observed differences in the gene expression patterns of DKK-3 (rs11544817) and CFH (rs10737680) highlight a possible regulatory association with disease status. These findings underscore the diagnostic potential of DKK-3 and CFH as biomarkers and risk indicators for both periodontitis and CAD. The modulation of their expression may represent a novel therapeutic strategy. However, further longitudinal studies are required to validate their prognostic significance and therapeutic applicability in disease management.

## Data Availability

The original contributions presented in the study are included in the article, further inquiries can be directed to the corresponding author.
